# Psychosocial Experiences of Front-Line Nurses Working During the COVID-19 Pandemic in Hubei, China: A Qualitative Study

**DOI:** 10.3389/fpubh.2021.780139

**Published:** 2022-02-16

**Authors:** Jianjian Wang, Yaping Zhong, Jinfeng Ding, Qiongni Chen, Jingjing Jiao, Chongmei Huang

**Affiliations:** ^1^Clinical Nursing Teaching and Research Section, The Second Xiangya Hospital, Central South University, Changsha, China; ^2^Department of Psychiatry, National Clinical Research Center for Mental Disorders, The Second Xiangya Hospital, Central South University, Changsha, China; ^3^School of Nursing and Midwifery, Monash University, Melbourne, VIC, Australia; ^4^Xiangya School of Nursing, Central South University, Changsha, China

**Keywords:** coronavirus disease 19 (COVID-19), front-line, healthcare provider, psychological experiences, qualitative study

## Abstract

**Background:**

A large number of nurses across China joined the anti-coronavirus disease 2019 (COVID-19) front-line in Hubei province, where the local healthcare system faced unprecedented challenges in the early 2020. Few studies have reported the psychological experiences of nurses from other regions who voluntarily participated in the response to the COVID-19 pandemic in Hubei province.

**Aim:**

To describe the psychological experiences of nurses who were involved in the anti-COVID-19 pandemic battle in Hubei province from January to April 2020.

**Methods:**

This was a qualitative descriptive study using purposive and snowball sampling strategies for participant recruitment. Twenty-four nurses were approached and twenty-one of them completed telephone interviews in April 2020. The interviews took an average of 75 min (range 34–140 min). Data were analyzed thematically after verbatim transcription of the interviews.

**Results:**

Our analysis generated three primary themes: (I) Contexts; (II) Psychological responses; and (III) Coping strategies (most participants identified suitable coping strategies including relaxing activities and seeking social support). Participants' psychological responses varied in four phases of the journey through the experience: (i) initiation phase: obligations and concerns/fears; (ii) transition phase: from overwhelmed to increased confidence; (iii) adaptation phase: a sense of achievement and exhaustion; and (iv) completion phase: professional and personal growth.

**Conclusion:**

Nurses had concerns, fears, and faced challenges working on the COVID-19 front-line. However, they were motivated by a strong sense of professional commitment. Most nurses successfully achieved personal and professional growth as they identified a range of coping strategies. Future research is needed to explore the long-term impact of the COVID-19 related working experiences on these nurses.

## Introduction

Millions of coronavirus disease 2019 (COVID-19) cases have been confirmed since it was declared as a public health emergency of international concern on January 30, 2020, by the WHO. As of 9 January, over 304 million confirmed cases and over 5.4 million deaths have been reported (1). This large-scale public concern exerts unprecedented pressure on healthcare systems and poses physio-psychological challenges to healthcare professionals. As of April 8, 2020, a total of 22,073 confirmed cases of COVID-19 have been reported among healthcare professionals in 52 countries, which aggravated the shortage of healthcare professionals. To alleviate the overwhelmed local healthcare system in Hubei, China, where the outbreak of COVID-19 was first reported, more than 42,600 healthcare professionals (including 28,670 nurses) from across China voluntarily went to Hubei for the fight against COVID-19 by April 7, 2020 (2). Earlier evidence has shown that a great majority of front-line healthcare professionals experienced clinically significant psychological symptoms (3–11). Healthcare professionals who traveled to the other regions in response to COVID-19 pandemic might have a greater risk of developing the aforementioned psychological symptoms due to their extra challenges relating to the entirely new working environment and social isolation (7, 12, 13). An understanding of their psychological experiences is essential to directly support them and to strengthen their capacity to provide a better care in the future healthcare crisis. However, only few studies have specifically focused on this group of healthcare professionals.

Nurses, women, and front-line healthcare professionals, and higher perceptions of risk of infection are the main associated factors for developing psychological symptoms relating to COVID-19 (3, 5, 10, 11, 14). These psychological problems could not only have a long-term effect on their wellbeing, but also decrease their work morality and efficacy, leading to hindering the urgent response to COVID-19 (8). Extant research has shown that healthcare professionals who experienced a more severe degree of psychological symptoms associated with disaster relief activities are more likely to report a job burnout and turnover (15–17). Compared to other healthcare professionals, nurses are more likely to perceive a high risk of infection and develop a post-traumatic stress disorder (PTSD) (10). To date, there are limited studies investigating their psychological experiences of involvement in pandemics, as distinct from other healthcare professionals, although nurses are the largest healthcare professional group and provide care to patients in the closest physical proximity.

Systemic reviews on the psychological impact of COVID-19 pandemics have indicated that front-line healthcare professionals, whose psychological symptoms were not identified and treated appropriately, could have maladaptive responses (e.g., PTSD) (18, 19). With its unpredictability and large infection scale, COVID-19 has the potential to have a deep psychological impact on healthcare professionals. Suicide has already been reported among front-line healthcare professionals providing care for patients with COVID-19 (20, 21). Front-line healthcare professionals can also have positive responses to the psychological impact of COVID-19 (e.g., post-traumatic growth) if they can adapt to the highly challenging events (7, 12). Understanding the psychological responses of the front-line nurses to the COVID-19 pandemic could inform the training, psychological support, and post-pandemic intervention for them. However, extant research has rarely reported the psychological responses of front-line nurses involved in the COVID-19 pandemic.

Therefore, this study aimed to describe the psychological experiences of nurses who were from the external provinces to Hubei and who were involved in the anti-COVID-19 pandemic battle in Hubei between January and April 2020.

## Methods

The study used a qualitative descriptive approach involving individual over-the-phone interviews based on the epidemic prevention and control policy. Qualitative description is widely adopted to gain knowledge from the key informants about poorly understood research questions in healthcare settings (22, 23). We considered this approach suitable for this study because we aimed to seek knowledge on the experiences of nurses involved in the combat against the COVID-19 pandemic.

### Sample and Setting

Participants were recruited by purposive and snowball sampling from the existing network of the authors. Eligible participants included nurses who were assigned from the other regions of China to work on the COVID-19 front-line in Hubei, China, between January and April 2020 for no <2 weeks.

Potential participants were approached by the authors *via* WeChat with an explanatory note already provided. Nurses, who were interested in the project, were invited to participate in an in-depth interview. An informed consent was obtained prior to the interview. We purposely selected participants from a balanced background, including gender, the level of education, and work roles and positions, to increase their representativeness.

### Data Collection and Analysis

All the interviews were conducted in Mandarin over the phone by three members of the research team in April 2020 and audio-recorded. A semi-structured interview guideline was first developed by the research team, and pilot tested on three nurses. The interview questions were subsequently adjusted based on the feedback from the pilot interviews. All the participants received the core questions 3 days before the interview to allow them to prepare.

The audio recordings of the interviews were transcribed in verbatim in Mandarin by the interviewer, and then proofread for accuracy by other members of the research team. The data were then thematically analyzed using Microsoft Excel in accordance with the Braun and Clarke's (24) six-step approach: (1) being immersed in data, (2) generating initial codes, (3) searching for themes, (4) reviewing themes, (5) defining and naming themes, and (6) reporting results. All participant information was de-identified prior to analysis. Data collection and analysis occurred concurrently until reaching saturation.

Two members of the research team, who were proficient in Mandarin, coded the transcripts independently. After the coding emerged, the researcher, who is proficient in Mandarin and English, translated all the codes and some primary extract quotes that illustrated the codes into English. An initial thematic map was developed based on the coding to form themes. Several meetings were held within the research team to discuss and to refine the themes (25).

### Ethical Consideration

Ethical approval was obtained from the Ethics Committee of Second Xiangya Hospital of Central South University in China on 25th Feb 2020 (NO.2020007). All participants provided either written or verbal informed consent prior to individual interviews. All interview recordings and transcripts were stored in an encrypted file which could only be accessed by the research team. Confidentiality was maintained throughout the research process.

## Results

### Participant Demographics

Twenty-four nurses accepted the invitation to participate in the study and three opted out for personal reasons. The remaining twenty-one nurses were interviewed individually (interview average duration 75.1 min; range 34–140 min). Among the 21 participants, 15 voluntarily joined the COVID-19 front-line and six were assigned by hospital authorities. The participants were aged between 25 and 42 years (mean age = 30.7). All the participants had more than 2 years of clinical experience in non-infectious departments (average 8.1 years; range 2.5–22 years), but seven of them had previous experience associated with infectious diseases. The duration they had served on the COVID-19 front-line ranged from 15 to 101 days at the time of the interview, and two participants were still on the COVID-19 front-line when their interviews were conducted (characteristics of participants are in Table 1).

**Table 1:** Characteristics of participants (*n* = 21).

**Characteristics**	
Ages in years, mean ± SD/median (range)	30.67 ± 4.88/30 (25–42)
**Gender**	
Female, *n* (%)	16 (76.2%)
Male, *n* (%)	5 (23.8%)
**Marital status and offspring**	
Married with children, *n* (%)	8 (38.1%)
Married without children, *n* (%)	5 (23.8%)
Unmarried without children, *n* (%)	8 (38.1%)
**Level of education**	
College, *n* (%)	2 (9.5%)
Undergraduate, *n* (%)	16 (76.2%)
Master, *n* (%)	3 (14.3%)
**Working experience in years**	
Mean ± SD/median (range)	8.07 ± 5.60/7.00 (2.5–22)
**Previous experience of infectious disease**	
No, *n* (%)	14 (70%)
Yes, *n* (%)	7 (30%)
**Duty on COVID-19 ward**	
Head nurses	2
General nurses	19
Days worked during on COVID-19 ward mean ± SD and median (range)	55.1 (15–101)

### Primary Results

Three main themes with four subthemes were obtained: (I) Contexts; (II) Psychological responses; and (III) Coping strategies as follows: (i) self-care: relaxing activities; (ii) peer support: comradeship; and (iii) family support. Participants' psychological responses varied in four phases of the journey through the experience: (i) initiation phase: obligations and concerns/fears; (ii) transition phase: from being overwhelmed to increased confidence; (iii) adaptation phase: a sense of achievement and exhaustion; and (iv) completion phase: professional and personal growth.

#### Contexts: Concerted Efforts

Healthcare professionals from other regions of China joined the anti-COVID-19 front-line in Hubei province where the local healthcare system faced unprecedented challenges. All the participants perceived that the whole nation was making a concerted effort to combat against the COVID-19 pandemic. For example, the state medical insurance administration in China imposed a coverage of testing and treatments for COVID-19. The participants stated that the collective spirit of the nation demonstrated our country's capacity and determination to conquer COVID-19 and to protect the people from the infection.

…* Every province was transporting supplies to Wuhan, like Anhui province sent 300 tons of fresh vegetables to support Wuhan … [Wulinghongguang A company cited] said: “We produce whatever the people need, for example, facial masks, intelligent mobile temperature measurement vehicles, unmanned disinfection vehicles, and unmanned transport vehicles ” …. (P15)*

#### Psychological Responses

The participants' experiences of working on the COVID-19 front-line can be classified into four phases: (i) initiation phase: obligations, concerns, and fears; (ii) transition phase: from being overwhelmed to increasingly confident; (iii) adaptation phase: a sense of achievement combined with exhaustion; and (iv) completion phase: professional and personal growth.

##### Initiation Phase: Obligations, Concerns, and Fears

Most participants voluntarily participated in the combat against COVID-19 and perceived that it was their moral obligation to join the combat. As nurses, they felt morally obliged to “*heal the wounded and rescue the dying”*. As citizens, they felt that they should take on the responsibility of serving their people and their country whenever necessary. For example:

*It's (joining anti-COVID19 combat) an instinctive response as a nurse. We have made pledges to heal the wounded and rescue the dying. This is the pledge given by all of us...(P10)*

All the participants expressed concerns when they made the decision to join the COVID-19 front-line. Their concerns included lack of knowledge about the disease, risk of infection to self and significant others, and working in an unfamiliar environment, which increased the level of uncertainty among the participants. Feeling nervous and fear were common amongst the participants during this phase. One participant described:

*The fear of being infected had always been there. I felt that any of my friends, colleagues or families and myself had a risk of being infected…(P20)*

##### Transition Phase: From Being Overwhelmed to Being Increasingly Confident

In addition to the concerns and fears discussed above, participants felt overwhelmed in the early stage of anti-COVID-19 by unexpected situations. For example, many participants were panicked by the large number of patients waiting for treatment on the first day they started work. A number of participants reported being powerless and unready when they were confronted with the sudden death of their patients and the high levels of distress and fears experienced by their patients. Confirmed cases were isolated in designated wards. They had limited contacts with their families who may also be patients with COVID-19 or even die from it. Provision of psychological support and bereavement services for these patients were also challenging for many of these nurses.

*There was an elder patient in our ward. His wife died and his daughter was infected in this epidemic…I don't know how to help him or comfort him…(P6)*

Most participants reported that they were gradually becoming more confident with caring for patients with COVID-19 as they had more experiences and received training. The training provided participants with precautionary measures and standard operating procedures that had facilitated their work safety and sense of security. They have confidence in the function of the personal protective equipment and felt secure in it. Some participants also mentioned that their confidence was also increased because the problem with the shortage of personal protective equipment was quickly addressed.

*We spent a whole afternoon on learning and practicing the standard procedures of wearing and removing protective suits… The head nurse checked it (protective suit) for us before we entered the ward. I knew I was safe...(P18)*

They reported that medical supplies delivered from other regions in China including supportive individuals, industries and home hospitals quickly addressed the shortage of personal protective equipment. This increased their confidence and courage to combat COVID-19.

*In the early days, when we were in short supply of protective equipment, many people helped us, including raising funds, to buy protective equipment and endeavored to mail those to us through various approaches...(P12)*

##### Adaptation Phase: Sense of Achievement and Exhaustion

In this phase, most participants found that their efforts paid off as the transmission of coronavirus was gradually contained, which brought them a high level of sense of achievement and job satisfaction. Their efforts also resulted in gratefulness from patients and a harmonious and trusting nurse-patient relationship. Most participants enjoyed the significance and meaningfulness of their efforts and commitment, and some expressed their willingness for future participation.

*I felt that the situation was getting better when I saw the number of discharged patients increased every day, which made me feel happy … I feel privileged and proud to join this combat...(P15)*

Despite the job satisfaction, three participants, especially those who worked in the intensive care units reported being exhausted by the long-time intensive work. They repeatedly mentioned that the use of personal protective equipment substantially contributed to their workload.

*Injection and blood drawing were easy. But it took twice as much time to do these with personal protective equipment… My clothes got often wet after a shift, so I just wanted to lie down when I was back to the hotel room...(P6)*

In this circumstance, some participants reported problems with sleeping and irregular menstruation. Many of them wished to win this war with COVID-19 and return to a normal life as soon as possible.

*I hadn't had a period for more than 50 days when I was in Wuhan, which was a sign of great stress…(P9)*

##### Completion Phase: Professional and Personal Growth

Most participants reflected that they gained a tremendous professional and personal growth from this combat. For example, many of them acquired new nursing skills. For example, one nurse who had no experience in working in an infection ward or intensive care unit prior to the combat reported that she learned mechanical ventilation.

*I accumulated a number of skills and knowledge from this experience, such as how to prevent hospital-acquired infections… how to care for patients with dyspnoea...(P8)*

Some participants developed new strategies to provide better quality care for patients. One of the examples was surrogate family, a role played by a responsible nurse of patients for whom the family could not visit them given the high risk of infection. These surrogate families provided multiple support which included replenishment of daily necessities, provision of psychological support, acting as an intermediary between the patient and the physician, developing individualized rehabilitation plan, and post-discharge follow-up.

*We adopted an approach “surrogate family”, so that each patient had a particular relative who looked after his/her daily life as well as treatments...(P6)*

Nearly all the participants reviewed the values embedded in nursing practice and reaffirmed the meaning of the nursing profession. They reported that nurses' commitment to the combat against COVID-19 gained a growing public recognition of the nursing profession and further constructed their professional identity and pride. While nurses were considered as heroes, some participants perceived that they only did their job as a nurse.

*Through this anti-epidemic experience, I have a stronger sense of nursing professional identity than I used to. 90% of the first-line healthcare providers are nurses, and it is us who spend the most time with patients...(P16)*

Some participants had no experience working in infectious wards or intensive care units and reported that they gained several professional expertise through this combat. They also reported that their communication ability was improved when they collaborated with team members from different specialties or different organizations.

*This is a new team, most of us are from different departments in our hospital and we are also some nurses of [hospital name] … I gradually learn how to facilitate effective communication within the interprofessional team...(P4)*

Personal growth was also commonly reported which mainly included strengthened willpower and realization of the importance of health and family. Many of them highlighted that they would hold a more positive attitude toward the future life.

*It is my first time facing so many deaths. I feel that life is so precious...It is really important to enjoy the life and the moment...(P13)*

#### Coping Strategies

Participants actively adopted various coping strategies to relieve stress, including: (i) self-care: relaxing activities; (ii) peer support: comradeship; and (iii) family support. They reported that they successfully adapted to their work environment and did not seek for psychological counseling, although this service was available from a number of agencies *via* hotline assistance.

##### Self-Care: Relaxing Activities

Relaxing activities were commonly adopted for self-care, such as singing, watching films, reading, doing exercise, and writing a diary. Three participants reported that they learned stress-relieving techniques online. Some participants intentionally avoided receiving information about COVID-19 after work to ensure a high-quality rest.

*I had kept the habit of writing diaries which had helped to sort things out and deal with things objectively…(P10)*

##### Peer Support: Comradeship

Participants predominantly reported that mutual support and encouragement among colleagues made the journey easier, and many regarded their colleagues as comrade-in-arms. They identified that talking with people who shared common experiences could effectively relieve work pressure. In addition, support and suggestions from colleagues who had experienced pandemics could help overcome the feeling of fear.

*We helped each other get dressed in the ward… On the shuttle bus, we often sang together… Some funny colleagues told us jokes… Everyone was trying to create a relaxing atmosphere…(P7)*

##### Family Support

Many participants reported that their family supported their engagement in the anti-COVID-19 pandemic battle even if their families were concerned about their safety. They identified that sharing adequate information about COVID-19 in Wuhan and sharing their work experiences with families was a good approach to relieving family concerns. They reported that family support enabled them to focus on patient care. However, two of them chose to hide the fact that they were involved in the combat from their parents.

*I had video calls with my parents every day and told them about my work and the situations in Wuhan. My parents were assured that I was safe there. For me, talking was a great way of relaxing...(P11)*

## Discussion

The COVID-19 pandemic has posed tremendous challenges to healthcare providers, especially to front-line nurses. They risked their lives to deal with the unpredictable situation. This qualitative study reported on the psychological experiences of front-line nurses from other regions of China who voluntarily participated in response to the COVID-19 pandemic in Hubei province. The results indicated that concerted efforts of the whole country and a strong sense of professional commitment motivated nurses to participate in the battle on the COVID-19 front-line. Their psychological responses generally experienced four phases from being full of obligation and having concerns and fears at the initiation phase, experiencing a mixture of overwhelming/exhaustion, and increasing confidence/fulfillment at the transition and adaptation phases, to achieving professional and personal growth at the completion phase. We also identified a range of coping strategies adopted by nurses to cope with psychological distress, such as relaxation activities, peer encouragement, and family support.

This study identified contexts that existed throughout all phases. It demonstrated that external factors contributed to the motivation of voluntary participation and positive experience of nurses in the fight against the COVID-19 pandemic. The whole nation united to combat the pandemic that alleviates psychological conflicts issue of the front-line nurses, such as the conflicts between fear and obligation (26). We found that lack of knowledge and skills, unfamiliar work environments and contents, concerns for family members and friends, powerless feelings, and difficulties in accepting the sudden death of patients were the common sources of concerns and stress among the front-line nurses (27), this is similar with As. As' study demonstrated that there are several sources of distress, ranging from fears of COVID-19 transmission, clinical challenges, and perceived lack of control, to concerns about family and home life (11). But the strong national backup force motivated them to go to Wuhan without hesitation, which is in line with our findings that nurses in this study more commonly had positive experience than negative experience, and this is consistent with Liu et al. (7).

This study identified four dynamic phases of the psychological experience of front-line nurses who voluntarily participated in response to the COVID-19 pandemic in Hubei province from other regions. Of the four phases, the phase of adaptation was not fully revealed in previous studies. The four phases of psychological experience were not always sequential, and some experience may be presented across multiple phases. For example, the sense of responsibility and concerns exist in all the four phases, and the sense of exhaustion occurred in both the adaption and completion phase but the specific features varied. This study also illustrated the gradual decrease of negative psychological experiences and increase of positive psychological experiences of nurses with their time of involvement grew, consistent with a previous study, the psychological experiences of anti-COVID-19 nurses in Hubei evolved perception such as obligations, concerns, fears at the initial stage of anti-COVID-19 pandemic (28). Similarly, a previous study found that anxiety was prevalent among health care staff during the MERS-CoV epidemic (29). Nurses in our study reported that they had concerns about working conditions, such as lack of adequate protective equipment and knowledge. Our findings of the study are supported by other research which declared that front-line healthcare providers have felt negative and complex emotions in the earlier stage of the pandemic (30). As the nurses received more training and support, they gained more experience and confidence. Through the results of this study, it is necessary to establish an effective communication and support mechanism for front-line nurses, such as the allocation of adequate protective devices and materials, and humanistic care for the families of front-line nurses, especially, there are vulnerable groups in their family, such as the elderly, children, and the disabled. group. Our study demonstrates that self-adjustment is useful in coping with stress, and an increasing psychosocial support is an important way to support the patients, which is congruent to a previous study (31).

In order to better support the patients and help them establish confidence in overcoming the disease, a number of front-line nurses play the role of “temporary family member” to their patients. This role required nurses to care for patients as both healthcare professionals and family members, including use of medicines, psychological counseling, health education, rehabilitation guidance, and support of daily living activities. The role of temporary helped nurses strengthen humanistic care, awaken the power of love, empower patients and health providers' courage to fight against COVID-19. For the majority of nurses, fears and concerns in the initiation phase were gradually replaced with positive perceptions and attitudes in the transition phase. In the adaptation phase, several front-line nurses also feel fatigued and overworked but also gain increased job satisfaction and a sense of achievement.

Our study showed that front-line nurses used various coping strategies to relieve stress. After the outbreak of the epidemic, the medical staff actively fought on the front line, but they are also faced with a heavy workload and psychological burden. Firm comradeship established which dispel the fear of COVID-19; WeChat workgroup communicates in real-time, timely answer doubts, give rapid treatment, relieve the anxiety of healthcare providers, and enhance their confidence in work. Furthermore, based on our results, family support helped nurses to fight against stress and fear. Similarly, it has been indicated that nurses have received psychosocial support from the government, from social environment, and from their families during the COVID-19 process and that they have expressed their emotions and feelings regarding the epidemic by keeping diaries (8). Therefore, a sound social support system for nurses should be urgently called for an establishment. The needs of the front-line nurses should be cared for by the government and by the society, including psychological and living needs and living needs. It follows that social and peer supports are crucial to promote the positive psychological experience of front-line nurses.

As the pandemic occurs abruptly, and the life of the majority of the population has been affected worldwide, the result of our study is the first study that specifically focused on the nurses who were the first wave of the anti-COVID-19 heroes in the rescue of the first epidemic center—Hubei province, so understanding of their psychological responses will facilitate a better psychological preparation of future epidemic rescue team.

However, this study has some limitations. It may not be able to represent the entire population of the front-line nurses in this combat, and, therefore, limit the generalizability of our findings. Additionally, most nurses were interviewed after completing their rescue tasks, whose responses to our questions were likely to be different from those who were still in fight against COVID-19 because of the environment change and recall bias.

The interviewee recruitment methodology of “looking for interview volunteers” might result in selection bias toward the nurses who coped better with the situation. While the interviews had been conducted to the observed data saturation, our findings can well-represent the situation of the involved nurses and explore the psychological experiences and coping strategies of the front-line nurses in more details for the future, and a larger sample research laid a good foundation. In this study, we did not obtain an additional qualitative information from the nurses; in the future, researchers should consider that, in order to have richer descriptions.

## Conclusion

Nurses had concerns, fears, and had faced challenges working on the COVID-19 front-line. This study generated three primary themes: (I) Contexts; (II) Psychological responses, and (III) Coping strategies. Participants' psychological responses varied in four phases of the journey through the experience: (i) initiation phase, (ii) transition phase, (iii) adaptation phase, and (iv) completion phase. Nurses were motivated to be engaged in an anti-epidemic work because of their strong sense of obligation, although they had concerns, fears, and had faced challenges working on the COVID-19 front-line. A range of coping strategies was identified to deal with these challenges, most nurses successfully achieved personal and professional growth. Future research is needed to explore the long-term impact of the COVID-19 related working experiences on these nurses.

## Data Availability Statement

The original contributions presented in the study are included in the article/supplementary material, further inquiries can be directed to the corresponding author/s.

## Author Contributions

JW, YZ, JD, QC, JJ, and CH made substantial contributions to conception and design, acquisition of data, analysis, and interpretation of data. JW, YZ, and JD involved in drafting the manuscript or revising it critically for important intellectual content. JW, YZ, QC, JJ, and CH given final approval of the version to be published and should have participated sufficiently in the work to take public responsibility for appropriate portions of the content. JW, JJ, and CH agreed to be accountable for all aspects of the work in ensuring that questions related to the accuracy or integrity of any part of the work are appropriately investigated and resolved. All authors contributed to the article and approved the submitted version.

## Conflict of Interest

The authors declare that the research was conducted in the absence of any commercial or financial relationships that could be construed as a potential conflict of interest.

## Publisher's Note

All claims expressed in this article are solely those of the authors and do not necessarily represent those of their affiliated organizations, or those of the publisher, the editors and the reviewers. Any product that may be evaluated in this article, or claim that may be made by its manufacturer, is not guaranteed or endorsed by the publisher.
